# Analyzing sociodemographic factors amongst blood donors

**DOI:** 10.4103/0974-2700.58667

**Published:** 2010

**Authors:** Namgay Shenga, KR Thankappan, CC Kartha, Ranabir Pal

**Affiliations:** IIDD Cell, Government of Sikkim, Sikkim, Sree Chitra Tirunal Institute for Medical Sciences and Technology, Thiruvananthapuram, Kerala, India; 1 Achutha Menon Centre for Health Science Studies, Sree Chitra Tirunal Institute for Medical Sciences and Technology, Thiruvananthapuram, Kerala, India; 2 Department of Cellular and Molecular Cardiology, Achutha Menon Centre for Health Science Studies, Sree Chitra Tirunal Institute for Medical Sciences and Technology, Thiruvananthapuram, Kerala, India; 3 Sikkim Manipal Institute of Medical Sciences, 5^th^ Mile, Tadong, Gangtok, India

**Keywords:** Education, occupation, religion

## Abstract

**Introduction:**

Blood transfusion is a fundamental and requisite part of any National Health Service for optimum management of emergency conditions like severe trauma shock and resuscitation with the optimum stock of its different components. The objective of the present study was to analyze the factors of knowledge of prospective blood donors that may influence their perception and awareness about blood donation.

**Materials and Methods:**

This population-based cross-sectional study was conducted at Gangtok in the state of Sikkim, India, on 300 subjects of the adult population selected by two-stage cluster sampling. The main outcome variables were the socioeconomic and demographic variables of knowledge of blood donation. By interview technique, using the pre-tested structured close-ended questionnaire, the principal investigator collected the data.

**Results:**

In our study population, 46% of the study population was found to have a high knowledge score. The knowledge about blood donation was found to be statistically significant with the occupational status and the education levels, both in the bivariate and in the multivariate analyses. Knowledge about blood donation was not significantly related to age, sex, marital status, religion, community status and per capita monthly family income.

**Conclusion:**

The study suggested that the perceptions toward voluntary blood donation could be influenced to a large extent by sociodemographic variables of knowledge among the general population.

## INTRODUCTION

The need for blood is an important concern to the society as a whole. Blood transfusion is a fundamental and requisite part of any National Health Service for optimum management of emergency conditions like severe trauma shock and resuscitation with the optimum stock of its different components, semantics. To run a hospital without a blood transfusion service is to invite severe difficulties in the daily operations of that institution. The reality is that no hospital can function effectively without an efficient blood supply.[1]

There should be enough blood and the component units in a blood bank available for everybody's requirements. The therapeutic use of specific portions – components of blood, e.g. factor VIII concentrates, packed red cells or platelets rather than whole blood is important in the advent of modern transfusion services. But, non-availability of sufficient basic blood units is a problem in Sikkim. The hospitals rely on the relatives of a patient to donate the necessary blood as there are not enough voluntary blood donations to help the needy patients. The blood is donated to a maximum on replacement basis. Blood banks stress the doctors, the nurses and the relatives of the patient and urge them to send replacement donors to maintain their stock. This is not a good system as the relatives of the patients are pressurized to find donors and it is frequently observed that professional blood donors are brought to donate blood in guise of being replacement donors. And, it is a well-established fact that professional donors constitute a group with high-risk behavior leading to greater chances of transfusion-transmitted diseases. The voluntary blood donation system in the state of Sikkim is 15%.[2]

Over a million blood units are collected from donors every year; nevertheless, many more millions still need to be collected to meet the global demand and ensure sufficient and timely provision of blood.[3]

Human blood is an essential element of human life and there are no substitutes.[4]

Voluntary donation system is by far the best and it needs to be strengthened. Thus, the study of understanding the various factors that could change the perception and awareness about blood donation among the general population may prove to be useful for the successful implementation of a blood donation program in the state, especially in improving the voluntary blood donation system. Blood donation is important for not only saving people's lives but also for the pursuit of a better social and living environment, and voluntary blood donation is of great social importance. It is important that voluntary non-remunerated blood donations should be promoted, accepted and practiced for safe blood supply.[5]

The objective of the present study was to identify the sociodemographic correlates for knowledge of prospective blood donors among the general population in Sikkim to understand the various factors that can help to plan the change in the perception and awareness about blood donation programs in the state. Broadly, there are three types of knowledge models: mathematical models, descriptive models and graphical models. Typically, a model only includes those variables that are sufficient to represent the phenomena in question. All models should be treated with caution. They are useful so long as the underlying assumptions are explicit and it is recognised that they are an abstract representation of reality that may, or may not, be objective.[6] Models in the social sciences tend to be descriptive and graphical rather than mathematical, although mathematical models have their place. In the knowledge management literature, almost all models are descriptive and graphical. Much of the academic literature deals with high-level abstractions that are presented as knowledge management models but are actually models of knowledge.[7] So far, there had been no study performed in this field in this state of India.

## MATERIALS AND METHODS

### Selection and description of the participants

This population-based cross-sectional study was conducted between 01.01.2004 and 30.03.2004 in the hill town of Gangtok in the state of East Sikkim, India, without any interventions. Three hundred adults between the ages of 18 and 55 years were selected as the study population. The list of the study population was taken from the electoral roll of 2002 Sikkim, 31-Gangtok Assembly Constituency from the Office of the District Collectorate of East Sikkim, with a total of 12,199 voters, assuming that at least 95% of the target population was included in the electoral list of this area.

The recruitment of 300 adults for the study was carried out by a two-stage cluster sampling technique. There were 15 polling stations under the Gangtok Assembly constituency with a total of 12,199 voters. Each polling station had a total of 500–1200 voters. In the first stage, 10 polling stations were selected randomly through a draw of lots. In the second stage, a total of 30 adults were randomly selected from each polling station.

Main outcome measures were socioeconomic and demographic variables of knowledge of blood donation, viz. age, sex, marital status, occupation, education, religion, community status and per-capita income.

### Content validity and reliability of study instruments

A pre-tested close-ended questionnaire contained 10 questions relating to knowledge about blood donation and its importance related with the sociodemographic situation prevailing in India. This was developed on the information provided by the global experts prior to the study for ensuring feasibility, acceptability, time management, validity and reliability at the institute with assistance from the faculty members and other experts. By initial translation, back-translation and re-translation followed by pilot study, the questionnaire was custom-made for the study. The interview schedule had two parts. The first part of the interview schedule was on socioeconomic and demographic characteristics. This included the variables age, sex, marital status, occupation, religion, community status and literacy status and per-capita monthly income. The second part of the interview schedule was on the knowledge about blood donation. The pilot study was carried out at the central blood bank of STNM Hospital among blood donors and non-donors, following which some of the questions from the interview schedule were modified.

### Data collection procedure

The house number was matched with the name and the serial number of the adult subjects. The house number was traced and then the subject randomly selected was identified for the findings of the study. The permission to conduct the study in Gangtok and STNM Hospital was taken from the Office of the Secretary Health and Family Welfare Department, Government of Sikkim. All the participants were explained about the purpose of the study and were ensured strict confidentiality. The verbal informed consents were taken from each of them before the interview. The participants were given the options not to participate in the study if they wanted. By an interview technique, the principal investigator collected the data. On an average, five to six interviews were conducted in a day. Details of the questionnaire can be provided, if required. Information on knowledge toward blood donation was disseminated in intensive health education sessions to complement the findings of the study to effectuate changes in willingness to donate.

### Statistical analysis

Bivariate and multivariate analysis was performed using MS Excel. From 10 questions relating to knowledge about blood donation and its importance, a summary indicator was later calculated giving scores to the questions, with due weightage being given to correct answers. The 33.3^rd^ and 66.7^th^ percentiles were used to categorize the subjects into three groups of poor, moderate and high knowledge.

## RESULTS

In 300 adults, 32% of the respondents had a poor knowledge score about blood donation and its importance while 22.0% had a moderate knowledge score and 46.0% were found to have a high knowledge score [Table 1].

**Table 1:** Knowledge scoring

Knowledge score	Number	Percentage
Poor	96	32
Moderate*	66	22
High**	138	46
Total	300	100

* MODERATE KNOWLEDGE SCORE: 33.3^RD^ PERCENTILE

** HIGH KNOWLEDGE SCORE: 66.7^TH^ PERCENTILE

The knowledge about blood donation was found to be statistically significant with occupation (*P*<0.005) and education (*P*<0.001) in the bivariate analysis. It was found that employed respondents had a higher knowledge score than the others and that there is an increase in the level of knowledge with an increase in the level of education [Table 2].

**Table 2:** Knowledge about blood donation and sociodemographic characteristics with which it was found to have a significant association in bivariate analysis

Socio demographic variable	Knowledge about blood donation			Number (%)	Chi-square (*P* value)
		
	Poor	Moderate	High		
Occupation					
Unemployed	26 (53.1)	10 (10.4)	13 (26.5)	49 (16.3)	
Office goers	28 (22.8)	25 (20.3)	70 (56.9)	123 (41.0)	
Businessman	29 (33.0)	20 (22.7)	39 (44.3)	88 (29.3)	0.005
Others	13 (32.5)	11 (27.5)	16 (40.0)	40 (13.3)	
Total	96 (32.0)	66 (22.0)	138 (46.0)	300 (100)	
Education					
Illiterate	12 (85.7)	2 (14.3)	None	14 (4.7)	
Primary school completed	24 (75.0)	3 (9.4)	5 (15.6)	32 (10.7)	
Junior school completed	23 (35.4)	17 (26.2)	25 (38.5)	65 (21.7)	<0.001
Secondary school completed	19 (19.8)	20 (20.8)	57 (59.4)	96 (32.0)	
Senior secondary and above	18 (19.4)	24 (25.8)	51 (54.8)	93 (31.0)	
Total	96 (32.0)	66 (22.0)	138 (46.0)	300 (100)	

The dependent variable, knowledge about blood donation, was taken into consideration in the multivariate analysis. Multiple logistic regressions were the preferred method taking into consideration that the dependent variables to be modeled were binary variables. The variables that were found to be significant in the bivariate analysis were also analyzed in the multivariate analysis. A significant relationship was observed with occupation and education in the multivariate analysis. Compared with the unemployed group, the employed group was found to have a greater knowledge about blood donation (odds ratio [OR] 2.49; 95% confidence interval [CI] 1.16–5.36; *P*<0.05). Similarly, the knowledge about blood donation was also found to be greater among the higher educated than the lesser-educated ones (odds ratio 1.69; 95% CI 1.33–2.15; *P*<0.001). In the multivariate analysis, knowledge about blood donation was 1.7 times higher among those with an education level more than junior school compared with less than junior school (OR 1.7; 95% CI 1.3–2.16). Compared with the unemployed, the employed respondents had 2.5 times greater knowledge about blood donation (OR 2.5; 95% CI 1.7–5.4) [Table 3].

**Table 3:** The results of multivariate analysis: Variables associated with knowledge about blood donation

Variables	Odds ratio	95% confidence interval	*P* value
Occupation			
Unemployed	1		
Employed	2.499	1.165–5.362	0.019
Businessman	2.054	0.931–4.533	0.075
Others	1.775	0.700–4.499	0.227
Education			
<Junior school	1		
≥ Junior school	1.699	1.336–2.159	<0.001

Knowledge about blood donation was not significantly related to age, sex, marital status, religion, community status and per-capita monthly family income [Table 4].

**Table 4:** Knowledge score with different sociodemographic variables, which was found to be not significant in the bivariate analysis

Sociodemographic variable	Knowledge about blood donation (no./%)			Number	Chi-square (*P* value)
			
	Poor	Moderate	Good		
Age group					
<30	26 (31.3)	11 (13.3)	46 (55.4)	83	0.060
30–39	37 (28.0)	36 (27.3)	59 (44.7)	132	
≥40	33 (38.8)	19 (22.4)	33 (38.8)	85	
Total	96 (32.0)	66 (22.0)	138 (46.0)	300	
Sex					
Male	69 (30.7)	51 (22.7)	105 (46.7)	225	0.681
Female	27 (36.0)	15 (20.7)	33 (44.0)	75	
Total	96 (32.0)	66 (22.0)	138 (46.0)	300	
Marital status					
Married	74 (31.1)	56 (23.5)	108 (45.4)	238	0.445
Single	22 (35.5)	10 (16.1)	30 (48.4)	62	
Total	96 (32.0)	66 (22.0)	138 (46.0)	300	
Religion					
Hindu	51 (32.1)	38 (23.9)	70 (44.0)	159	0.551
Buddhist	38 (33.6)	24 (21.2)	51 (45.1)	113	
Others	7 (25.0)	4 (14.3)	17 (60.0)	28
Total	96 (32.0)	66 (22.0)	138 (46.0)	300	
Community status					
Schedule tribe	36 (35.6)	20 (19.8)	45 (44.6)	101	0.264
Schedule caste	4 (20.0)	8 (40.0)	8 (40.0)	20	
OBC	35 (32.1)	27 (24.8)	47 (43.1)	109	
Others	21 (30.0)	11 (15.7)	38 (54.3)	70	
Total	96 (32.0)	66 (22.0)	138 (46.0)	300	
Per-capita monthly income					
<700	31 (26.3)	27 (22.9)	60 (50.8)	118	0.454
700–1100	30 (38.0)	15 (19.0)	34 (43.0)	79	
>1100	35 (34.0)	24 (23.3)	44 (42.7)	103	
Total	96 (32.0)	66 (22.0)	138 (46.0)	300	

## DISCUSSION

Our observation regarding the disparities in blood donation behavior and to understand the problems to improve voluntary blood donation revealed that 46% had a high knowledge score and was related statistically significantly with the occupation and the education. There was no comparable study in the Indian subcontinent on analysis of factors related to knowledge of blood donation.

The Baltimore study showed that low rates of voluntary blood donation by the general public have been attributed to a variety of socioeconomic, medical and attitudinal factors. Lack of awareness of the need for donation, fear of donating blood related to perceived risk of contracting human immunodeficiency virus and loss of physical vitality after donation have been proposed as potential reasons for ethnic and racial disparities in blood donation.[8]

There was no statistically significant association of knowledge about blood donation with sociodemographic indicators like age, sex, religion and community status and per-capita monthly income. Comparable observations were also found among the students of Chulalongkon University, Thailand, where 80% of the participants knew about blood donation and 11% of the study population (n = 400) had even donated blood voluntarily. The study, however, did not find any significant correlation of gender, age and educational level with knowledge about blood donation. The findings of the study concluded that greater knowledge about blood donation does not lead to donation and that specific campaigns are needed to convert this into actual voluntary donation.[9]

The current results were in agreement with one study in the United States where it was found that educational level and family income are both strong indicators of the probability of someone's donating blood.[10] Studies identified that among demographic groups the primary reason to donate blood was altruism (i.e., a general desire to help others such as friends, relatives or those affected by disaster). However, knowledge and awareness of the need for blood in the community was an important motivating factor. The response observed after a national disaster demonstrates that, in those situations, the donors perceive that the benefits associated with giving blood (e.g., knowing that one has helped those affected by a disaster or has performed his or her civic duty) clearly outweigh the costs (i.e., the fear or inconvenience).[11–13] One step in that direction is to ensure that the public and the media are aware of the continuous need for blood and the importance of donating regularly.[14]

The study of understanding the various factors that could change the perception and awareness about voluntary blood donation among the general population may prove to be useful for improving the blood transfusion services in the state. We recommended that health education for removal of myths and misconceptions along with a provision of better transfusion services will help us in the long run to reach the necessary supply of blood in this state of India by encouraging more people to become regular and voluntary donors.

### Strengths of the study

The study's strength lies on dealing with a novel idea, which was a significant and important issue. We conducted the study to analyze the factors that may influence blood donation, which is a component of stockpiling and usage for various emergency conditions like trauma, shock and resuscitation. There had been a number of theoretical models that would acknowledge that ‘knowledge’ is an important contribution to action – in this case, potentially donating blood. Knowledge domains of blood donation were assessed because the lack of knowledge could be an important barrier. The variables being studied will directly impact the amount of blood donated; thus, influencing the amount of blood available for patients of trauma and other indications.

### Limitations of the study

This study contained a new and unique idea that evaluated correlations between overall knowledge on the blood donation and donor demographics that may influence inventory and usage of blood for various emergency conditions. The study could not be developed later to understand the reason behind this disparity in knowledge on voluntary blood donation due to resource constraints.

### Future directions of the study

Research on voluntary blood donation had been performed globally for decades to understand better donation efficiency, safety, retention, collection numbers and diversity of the donor philosophy. The need for blood was growing day by day as a result of advancement in the medicine. Blood was always in short supply and recruitment of voluntary donors was never met fully. Adoption of innovative techniques for the recruitment of voluntary blood donors will motivate people at large. Considering the accelerated demand for blood, it was surprising that so little research had been performed in this field. In most of the studies conducted in this field, it was found that people tended to stress fears, possible ill effects along with different types of myths and misconceptions about blood donation to conclude that an appropriate motivational campaign be launched to increase voluntary blood donation.

Investigations were also needed to assess research directions to develop well-designed interventions. New regulatory requirements for donor eligibility challenge blood centers to recruit and retain enough donors. Moreover, donor satisfaction varied among demographics and was positively correlated with the intent to return for future donation. The findings in a majority of the studies suggested that the primary motivation among all donors was altruism; incentives to future donation may need to be tailored according to demographic variables. Modern sources of positive and negative impetus were worth exploring through scientifically sound studies using multipronged approaches. Strategies that focus on recruitment and retain voluntary donors as well as transforming first-time donors into repeaters were needed. Investigations were also needed to assess research questions and to develop well-designed interventions to test hypotheses and to produce generalizable future observations. The researchers should have employed optimum methodology to assess the effects of donor knowledge and perceived risk on the intentions as many undue fears are associated with the inhibition to blood donation.

## CONCLUSION

The finding of the research indicated that the perceptions toward blood donation could be influenced to a large extent by knowledge significantly related with the occupation and education among the general population. The regular flow of voluntary blood donors will have a cumulative effect on the different strata of society leading to a reduction in unnecessary fear associated with voluntary blood donation. Understanding blood donor motivations was crucial to improving effectiveness of donor recruitment and retention programs. The research concepts and techniques should have examined whether demographics mirror the donor pool to assist in targeted recruitment that actually leads to the factors influencing blood donation, which is a regular stock of blood components needed for the crisis like trauma, shock and resuscitation. We have to sincerely motivate prospective donors in the sociocultural setting, both inside the hospital as well as in the community.
